# Literature and Medicine

**DOI:** 10.1002/jhm.70026

**Published:** 2025-03-13

**Authors:** Aditi Mahajan

**Affiliations:** ^1^ Georgetown University Washington D. C. District of Columbia USA

In the 1960s sociologists observed that many physicians practiced medicine with “detached concern” and for decades this seemed to be the goal.^1^ Even now, in medical education, we are taught to care but not too much, taught to empathize but not take things home with us, taught to listen but not to absorb, but every day as I go to work and I rotate under a revolving door of residents, fellows, and attendings, the ones who I look up to the most, and the ones who seem to change their patients lives the most are the ones who engage beyond the science of medicine. As a third‐year medical student trying to understand what kind of doctor I want to be, I have been paying close attention to how medicine is being practiced.

A theme that has echoed throughout all of my rotations, especially on the outpatient side, was that patients repeatedly said that they “just wanted to feel like someone understood what they were going through”. These were patients who had healed physically, been sent home to loving family and friends, our hands washed of our role in their journey. But now, the patients were stuck with another mountain to scale, seemingly alone. They had to process the traumatic event of illness and of healing, and they felt so alone in that journey. One instance that stands out to me was when I rotated through Internal Medicine. I spent 1 month on inpatient wards, spending multiple days with patients, tracking every lab value, and checking in with them multiple times a day. Then I rotated through outpatient medicine clinics. In one of my cardiology outpatient visits, I met a patient who had recently been admitted to the hospital for hypertension and heart failure. She came to clinic feeling frustrated and overwhelmed. In talking to her she emphasized that while she was in the hospital people were paying a lot of attention to her; weighing her daily, tracking her labs, and keeping her on a cardiac sodium restricted diet. She got better and then was discharged home, where she lived alone with a dog to take care of. While she was happy to be home, she realized that she now had to manage her health herself. She alone was responsible for tracking her diet, checking her blood pressure, and taking her pills. It was a stark change from being admitted and she was struggling to adjust. The doctor I worked with was phenomenal and immediately reassured her and worked with her to set up plans for tracking her intake, taking her medications, and keeping in contact with the office. He also made it a point to connect her with a heart failure support group, providing her community and support during this difficult transition period. This doctor was wonderful and attentive and had the resources on hand to help her. After she left, he also took the time to show me how to navigate local support group resources and emphasized the importance of long‐term patient care. He shared that he had experienced some health difficulties a few years ago and ever since then had understood how to better support patients in the long term. His experience had changed how he practiced medicine. However, not everyone has experiences like that. Many doctors have been practicing for decades and know illnesses like the back of their hand but cannot understand what it feels like to experience them. Practicing medicine, while emphasizing narrative competence, pushes us toward a better relationship with our patients. It allows patients to unburden themselves from the mental strain of healing alone and symbiotically it allows us as practitioners to create a therapeutic alliance and develop a deeper understanding and relationship with each patient that we encounter.

Developing narrative competence is essential and like any other muscle in our body, our ability to elicit and share narratives must be practiced to grow. However, healthcare providers are already stretched thin with many competing demands and barriers to achieving narrative competence are plentiful and varied across specialties. Primary care physicians noted that patients were not always emotionally ready to talk to them, pediatricians cited trust as a large barrier, and surgeons emphasized logistics or patient data.^2^ One thing that remained consistent across all fields was a lack of time providers are able to spend with patients. Some possible solutions could be educating providers on time management strategies or exploring more time‐efficient strategies to eliciting patient narratives.^3^ However, so much of our schedules as students, residents, and providers are out of our control and more large‐scale systemic changes need to occur to grant patients more face time with their providers. In the interim, as students we can utilize our spare time to engage more deeply without our patients and by learning about narrative competence early and practicing our skills throughout our training, we can hopefully build it into our practice from the start. This approach requires discipline, community engagement, and consistent reflection on one's practice.

Many physicians have turned to studying literature to grow their personal understanding of narration and of illness. For physicians to read well written stories and to practice writing them, to polish their skills as readers, interpreters, and analyzers of the worlds of others, and to force themselves to sit in the discomfort of sharing their own story, literature can be used as a steppingstone to mastering narrative competence. Personal reflection, analysis through conversation, and vulnerability in sharing all lean on literature to take medicine to new heights.

In my second year of medical school, I joined a track offered at the Georgetown School of Medicine called Literature and Medicine. Literature and Medicine is a longitudinal 3‐year track focused on engaging students in the intersection of literature, narrative studies, medical narratives, and medicine. Led by Dr. Dan Marchalik, a Urologist and MA, the Literature and Medicine track or LitMed as its affectionally called by students has become a place for community and introspection. In creating the track, Dr. Marchalik had noted that students faced immense difficulty adjusting to different facets of medical education, including anatomy lab, the wards, and difficult patients. He noticed the burnout that was rampant and realized that a common denominator “appeared to be the student's inability to reflect on their own experience ‐ to maintain the power to create meaning”.^4^ Through this realization and the subsequent creation of LitMed, he utilizes fiction to inspire introspection. While the original graduating class of LitMed left Georgetown in 2017, the culture continues. In my time in the track, we have read more than 20 books. Each meeting is held on a Tuesday night, and I find myself ruminating over conversations we had well into the weekend.

One book that stands out to me still is Danya Kufafka's Notes on an Execution. This book is unnerving and intriguing. It follows a man who is sentenced to death for the murder of women in his life and forces us to consider a perspective we are not usually privy to. It asks us to understand the protagonist, and because we have access to his thoughts and his reasons, we kind of do. This book and its subsequent discussion had us all considering how we justify actions in our lives, how we challenge preconceived notions, and how we have to let go of judgment to understand people just a little better. Another story that stands out is Happiness Falls by Angie Kim. Completely different than Notes on an Execution, it is a story about mystery, communication, and the different ways we interact with the people and the world around us. Our conversations centered on communication, what we take for granted and what we must do to understand each other better. This book came at an opportune time for me, in my transition from preclinical learning to participating on the wards, and it forced me to take an extra moment at each bedside to invest in learning how to communicate with every patient.

These books, and these discussions allow us to sit in a room with our peers and challenge each other. We get to try to understand the author together, what message they want us to receive, and we get to decide what message we all chose to receive and how those messages differ from each other. I have walked into every LitMed meeting confident in my thoughts and perspectives and I have walked out of every LitMed meeting with unanswered questions filling my mind. Fiction, and LitMed, forces us to be flexible with our thoughts, allowing people to challenge us and our preconceived notions—a skill directly translatable to the hospital environment. It allows us to understand our patients better, to treat them as people and not just patients, and more than anything it allows us to remain human.

Over the last few months on my clinical rotations, I have made an active effort to use my extra time with patients to sit with them, ask them questions, and hear their stories. Not the one liners or assessments we've been trained to memorize but their illness and life course, with little mention of symptoms and focused primarily on their experience. I've been able to see firsthand the importance of narrative competence, defined as “the ability to recognize, absorb, interpret, and act on the stories of others”, be used in healthcare to help validate a patient's experience while also encouraging creativity and self‐reflection from us as providers.^5^

By focusing on the humanity of doctors and patients, we can understand each other better, trust each other more, and build a future together. By combining literature and medicine, we all become better equipped to weather the unpredictable storms of illness and disease.

## CONFLICT OF INTEREST STATEMENT

The author declares no conflicts of interest.
